# Expression and function of Siglec-15 in RLPS and its correlation with PD-L1: Bioinformatics Analysis and Clinicopathological Evidence

**DOI:** 10.7150/ijms.77193

**Published:** 2022-10-31

**Authors:** Lixuan Cui, Xiuyun Tian, Liang Yan, Xiaoya Guan, Bin Dong, Min Zhao, Ang Lv, Daoning Liu, Jianhui Wu, Chunyi Hao

**Affiliations:** 1 Key Laboratory of Carcinogenesis and Translational Research (Ministry of Education/Beijing), Department of Hepato-Pancreato-Biliary Surgery, Peking University Cancer Hospital & Institute, Beijing, People's Republic of China.; 2 Key Laboratory of Carcinogenesis and Translational Research (Ministry of Education/Beijing), Department of Critical Care Medicine, Peking University Cancer Hospital and Institute, Beijing, People's Republic of China.; 3 Key Laboratory of Carcinogenesis and Translational Research (Ministry of Education/Beijing), Central Laboratory, Peking University Cancer Hospital & Institute, Beijing, People's Republic of China.; 4 Key Laboratory of Carcinogenesis and Translational Research (Ministry of Education/Beijing), Department of Pathology, Peking University Cancer Hospital & Institute, Beijing, People's Republic of China.

**Keywords:** Siglec-15, PD-L1, Retroperitoneal liposarcoma, Prognosis

## Abstract

***Purpose*:** Retroperitoneal liposarcoma (RLPS) is a rare malignancy without effective treatment. Since current treatment for unresectable RLPS is unsatisfactory, immunotherapy and targeted therapy are urgently needed. Siglec-15 is a transmembrane protein highly homologous to PD-L1 and is involved in tumor immune escape. The biological function of Siglec-15 in RLPS, its prognostic relevance and its relationship with PD-L1 need to be further clarified. In this study, we aimed to explore the biological function of Siglec-15 in sarcomas through bioinformatics analysis, and we also evaluated Siglec-15 and PD-L1 expression in RLPS samples. The relationship between the expression of Siglec-15 and PD-L1 and their clinicopathological relevance and prognostic value were also investigated in clinical RLPS patients.

**Methods:** The RNA sequencing data of 259 sarcoma cases and 48 RLPS cases from TCGA were used to analyze the Siglec-15 expression and the differentially expressed genes (DEG) related with Siglec-15 expression. In addition, DEGs were subsequently analyzed through the gene ontology (GO)/ Kyoto Encyclopedia of Genes and Genomes (KEGG) and protein-protein interaction (PPI) network. Tumor specimens were obtained from 91 RLPS patients of our sarcoma center, and Siglec-15 and PD-L1 expression were evaluated using immunohistochemistry. The correlation between the expression level of these two markers as well as their correlation with clinicopathological factors and prognosis of RLPS patients was also assessed.

**Results:** GEPIA analysis showed that the high expression of Siglec-15 was associated with poor sarcoma OS (P=0.034). A total of 682 differential genes were identified between the high and low expression groups of Siglec-15 in RLPS. Enrichment analysis of the KEGG pathway showed that Siglec-15 was related to the Hippo signaling pathway and the neuroactive ligand-receptor interaction. GO annotation analysis showed that the expression of Siglec-15 may thus be able to affect serine hydrolase activity, alongside signal receptor activator activity. The top 5 genes with the largest number of connection points are APOA1, F2, AHSG, AMBP, SERPINC1. In subsequent studies, we used 91 liposarcoma samples from our center for verification. Siglec-15 was expressed in 84.6% of RLPS cases, whereas PD-L1 was expressed in 17.6% of RLPS cases. A negative correlation was observed between Siglec-15 and PD-L1 expression (*P*=0.020). In this group of RLPS patients, high Siglec-15 expression was correlated with poorer disease-free survival (DFS) (*P*=0.021), and it was an independent predictor of DFS (hazard ratio: 2.298; 95% confidence interval: 1.154-4.576; *P*=0.018). However, we did not find a correlation between PD-L1 expression and overall survival or DFS in RLPS patients.

**Conclusion:** The DEG and signaling pathways identified in the study could provide a preliminary understanding of the underlying molecular mechanisms of Siglec-15 in the development and progression of RLPS. High expression of Siglec-15 was a negative independent predictive factor for DFS of RLPS. The negative relationship between Siglec-15 and PD-L1 expression suggested that the Siglec-15 pathway might be an important supplement to PD-L1 treatment.

## Introduction

Retroperitoneal soft tissue sarcoma (RPS) is a rare malignancy with an estimated incidence of 0.5-1 per 100,000 people 1. Though there are over 70 pathological RPS types, retroperitoneal liposarcoma (RLPS) is the most common subtype accounting for 45% of all RPS cases 1, 2. Currently, surgical treatment is the most effective treatment for RLPS 3. However, treatment for recurrent and advanced sarcomas remains unsatisfactory 4-6, and multidisciplinary treatment is urgently needed for RLPS. DNA structure analysis suggests that different pathological types of sarcomas might have different immunological therapeutic potential 7, 8.

Cancer immunotherapy, including immune checkpoint inhibitors, adoptive cell therapy, and tumor vaccines, has shown promising effect in a variety of tumors. Clinical trials with immune checkpoint inhibitors have also been conducted for RLPS on a smaller scale, but in contrast to other tumor types such as lung cancer and gastric cancer, investigation on the use of immunotherapy for RLPS is progressing slower 9, 10. In these clinical trials, liposarcoma patients with positive response to immunotherapy were limited, suggesting that we still need to elucidate the function of immune checkpoints in RLPS and to determine more effective immunotherapeutic targets for managing RLPS 11.

The programmed cell death-1 (PD-1)/programmed cell death ligand-1 (PD-L1) pathway is one of the well-known pathways involved in immune evasion. Both malignant cells and immune cells can express PD-L1. PD-1/PD-L1 inhibitors can induce sustained remission in a variety of advanced cancer patients 12, 13. Therefore, the PD-L1 expression in tumor cells and the tumor microenvironment was of clinical significance 12. PD-L1 expression has also been used to predict prognosis and response to immunotherapy in gastric and lung cancer patients 9, 14. However, owing to heterogeneity and a small sample size, previous studies reported conflicting results regarding the predictive value of PD-L1 in patients with soft tissue sarcomas (STS) 15-17. Therefore, the clinical relevance and prognostic value of PD-L1 in RLPS needs further clarification.

In recent years, sialic acid-binding immunoglobulin-like lectin-15 (Siglec-15) has been found to play an important role in tumor immunity and may act as a potential target for immunotherapy 18-20. As a member of the sialic acid-binding immunoglobulin-like lectins, Siglec-15 can inhibit antigen-specific T cell responses 19. Siglec-15 has high structural homology with PD-L1 and a similar extracellular domain to the B7 family, indicating that it might have functions similar to B7 immunomodulatory molecules 18. The expression of Siglec-15 and PD-L1 in lung cancer is mutually exclusive, indicating that they might have different regulatory mechanisms 19. Currently, there is no report on the role of Siglec-15 in RLPS patients, so the function and mechanism of Siglec-15 in RLPS remains to be elucidated.

The analysis of high-throughput sequencing data from the public biology platform is a useful method to explore gene-expression differences and the related functional signaling-pathways, and to provide new therapeutic targets 21, 22. Therefore, we performed bioinformatics analysis on the next-generation sequencing results of sarcoma tissues stored within the TCGA database, so as to clarify the prognostic value and related biological functions of Siglec-15 in RLPS and the sarcoma.

In this study, we aimed to explore the role and potential mechanisms of Siglec-15 in sarcomas by bioinformatics analysis and to evaluate Siglec-15 and PD-L1 expression in our own RLPS cases. The relationship between the expression of Siglec-15 and PD-L1 and their clinicopathological relevance and prognostic value were also investigated in RLPS patients.

## Methods

### Data sources for sarcoma and retroperitoneal liposarcoma

The Cancer Genome Atlas (TCGA) is a free public bioinformatics database based network, which includes data of copy number variation and gene expression, methylation, and clinical prognostic information 23. As of August 2021, TCGA covers more than 84,000 tumor cases. We downloaded the RNA-seq data of 259 cases of sarcoma, and 48 cases of retroperitoneal liposarcoma from the "TCGA-SARC" project for subsequent analyses of the function of Siglec-15.

### Survival analysis of Siglec-15 in RLPS and sarcoma derived from TCGA

The GEPIA (Gene Expression Profiling Interactive Analysis) is a free web-based tool for online bioinformatics analysis of the TCGA database 24. Survival analysis was performed by GEPIA based on the Siglec-15 expression in sarcoma derived from TCGA. Median expression was used as a cut off value for the survival analysis. Log-rank tests were used for hypothesis evaluation. The survival plot includes hazards ratio based on Cox Proportional-Hazards Model and 95% confidence interval information. The DFS and OS of 48 RLPS patients were extracted from the TCGA database, and SPSS (software version 22.0; Chicago, IL, USA) was used for K-M survival analysis in RLPS.

### Identification of differentially expressed genes (DEGs)

We first generated an mRNA matrix from the downloaded RNA-seq data, and completed the overall gene name association conversion. According to the median count, tumor tissues were divided into Siglec-15 high expression group and Siglec-15 low expression group. We applied the R package V4.1.1 (13) to identify DEG, and calculated the logarithmic fold change (FC). The linear model based on the empirical Bayesian distribution was used to calculate the significant difference level (P value) 25, 26. Used the Benjamini‑Hochberg method to obtain the false discovery rate (FDR), FDR<0.05 and |log FC|>2.0 are used for specific cut-off criteria for DEG 27. Used the pheatmap package in R version 4.1.1 to generate a heatmap.

### GO function annotation and KEGG pathway enrichment analyses of Siglec-15 in RLPS and sarcomas

In order to further study the role of Siglec-15 in sarcoma, we use the enrichplot package in the R package to analyze the GO function annotations from three aspects of biological process, cell composition and molecular function. Meanwhile, the KEGG pathway enrichment analysis was performed to find the signal pathways closely related to Siglec-15.

### Construction of PPI network and screening of network core genes

Protein-Protein Interaction Networks Functional Enrichment Analysis database (String https://string-db.org/) was used in the construction of PPI network. The differential genes screened from different Siglec-15 expression groups are submitted to the string database for analysis. Used PPI pair with minimum interaction score of 0.9 to construct a PPI network. Core nodes are vitally important to ensure the stability of the entire network. According to the score of the number of core nodes with calculated by the String website, used R to draw a bar graph. In this experiment, the 30 genes with the greatest number of adjacent nodes were clarified and classified as the network core genes.

### Clinical samples and patients

All tumor samples used in this study were obtained from 91 RLPS patients who underwent surgical resection at the Sarcoma Center of Peking University Cancer Hospital (Beijing, China) between March 2009 and August 2019. Clinicopathological features and postoperative follow-up data were collected. RLPS was classified as well-differentiated liposarcoma (WDLPS), dedifferentiated liposarcoma (DDLPS), pleomorphic liposarcoma (PLPS), and myxoid/round cell liposarcoma (MLPS) according to the World Health Organization classification and graded according to the Federation Nationale des Centres de Lutte Contre le Cancer (FNCLCC) grading system 28, 29. Among the 91 cases, 61 were diagnosed as DDLPS, 21 as WDLPS, 7 as PLPS, and 2 as MLPS. All patients were followed up from June 2013 to July 2020. The median follow-up time for these patients was 39.7 months (range: 1.3-104.4 months). The average age of the 91 RLPS patients was 56.1 ± 11.3 years old. The detailed characteristics are also described in Tables 1 and 2. Patients who received chemotherapy or radiotherapy before surgery were excluded. All patients in this study signed a written informed consent. This study was approved by institutional review board of the Peking University Cancer Hospital (approval no. 2019KT19).

### Immunohistochemical assessment

Within 30 minutes of resection, the tumor samples were fixed in formalin and were embedded with paraffin for long-term storage. Five micrometers thick sections were baked at 60 °C for 2 h and then placed into xylene. Graded concentrations of alcohol (100%, 95%, and 80%) were used in the process of rehydration. Endogenous peroxidase in the samples was blocked with 3% hydrogen peroxide for 15 min, followed by rinsing with phosphate-buffered saline (PBS, pH 7.3; Beijing Zhongshan Golden Bridge Biotechnology Co., Ltd., China). A high-pressure antigen repair method was applied in our study. The specimens were placed in an EDTA antigen retrieval solution (pH 8.0; Beijing Zhongshan Golden Bridge Biotechnology Co., Ltd., China) for 2.5 min in a pressure cooker. Then, the slides were placed at room temperature for natural cooling followed by three rinsing steps with PBS. Goat serum (Beijing Zhongshan Golden Bridge Biotechnology Co., Ltd., China) was used to block non-specific staining for 1 h. PD-L1 rabbit monoclonal antibody (Beijing Zhongshan Golden Bridge Biotechnology Co., Ltd., China) and Siglec-15 rabbit monoclonal antibody (dilution 1:800; cat. no. NBP2-41162; Novus Biologicals, USA) were used as the primary antibodies. After incubation with the primary antibody at 4 °C overnight, the slices were rinsed three times with PBS to remove the unconjugated primary antibody. The primary antibody was bound by ready-to-use EnVision reagent (EnVision Detection System Peroxidase/DAB, Rabbit/Mouse; Dako, Denmark), then DAB (1:50, diaminobenzidine, Rabbit/Mouse; Dako, Denmark) was used for dyeing. Hematoxylin was used for counterstaining, followed by dehydration using graded alcohol and transparentization using xylene.

### Staining evaluation

PD-L1 and Siglec-15 expression in the samples were scored by two independent pathologists who were blinded to the clinical data of the patients. An immunoreactivity score (IRS) system was used in this study. The percentage of positive cells and the staining intensity determined the IRS, with scores of 0-1, 2-4, 5-8, and 9-12 evaluated as “-”, “+”, “++”, and “+++”. “-” was identified as negative, and “+”, “++”, and “+++” were identified as positive. In subsequent analyses, “-” and “+” were defined as low expression, whereas “++” and “+++” were defined as high expression.

### Statistical analysis

Correlations between immunochemical staining and clinicopathological parameters were assessed using the χ^2^ test and Fisher's exact test. The relationship between PD-L1 and Siglec-15 expression was analyzed using Pearson's χ^2^ test. Survival curves were generated using the Kaplan-Meier method, and the correlation between prognosis and PD-L1 or Siglec-15 expression was calculated using the log-rank test. A Cox proportional hazard regression model was used for univariate and multivariate survival analyses to identify independent parameters affecting overall survival (OS) and disease-free survival (DFS). When the *P* value was < 0.05, the result was considered significant. Statistical analyses were performed using SPSS (software version 22.0; Chicago, IL, USA).

## Results

### Survival analysis in RLPS and sarcoma patients collected from TCGA

**Figure 1:**
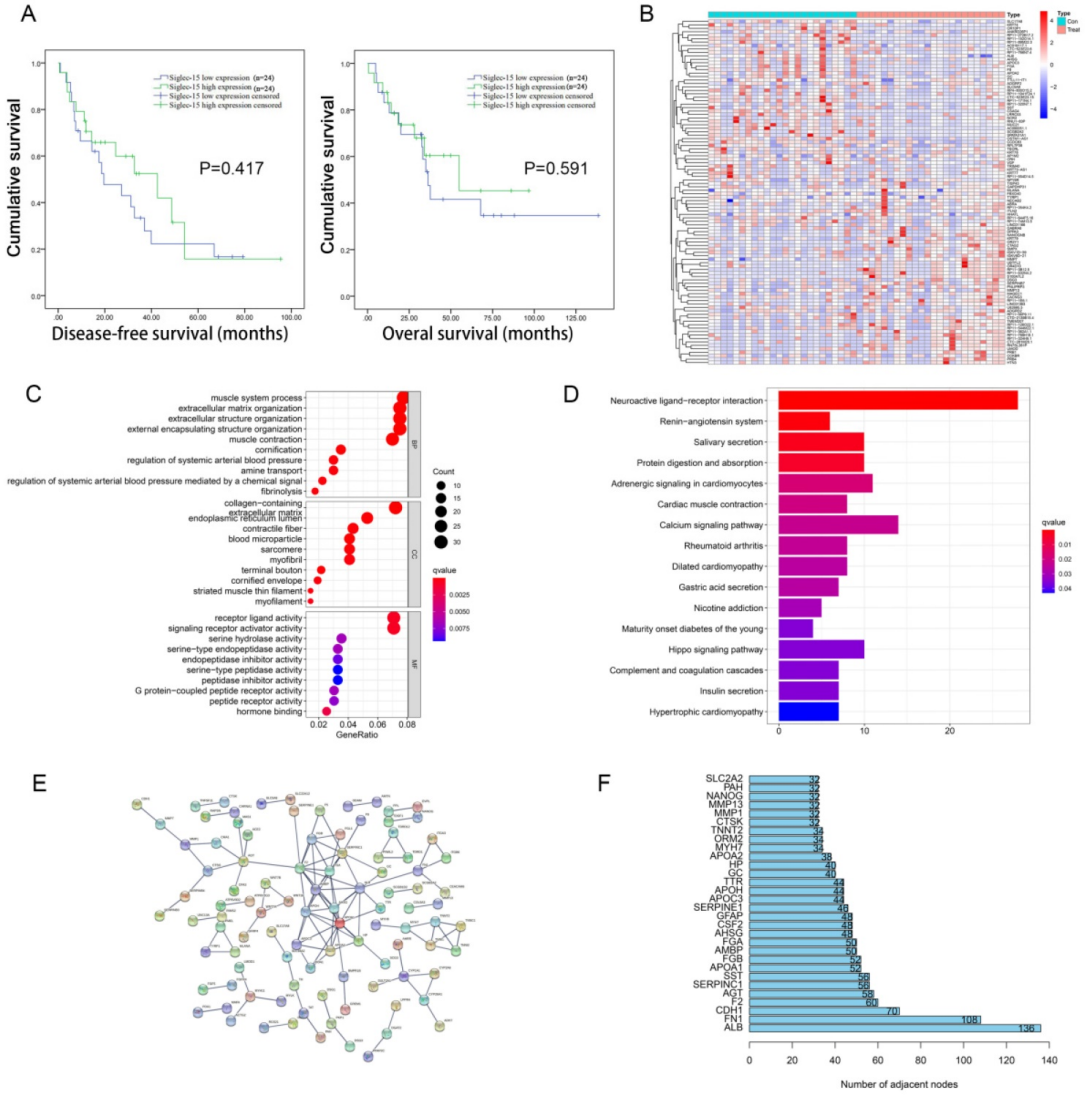
** Prognosis value and functional enrichment analysis of DEG in RLPS samples with low and high Siglec-15 expression. (a)** Survival curve of differential Siglec-15 expression were analyzed in 48 retroperitoneal liposarcoma patients. **(b)** Representative heatmap of DEG between Siglec-15 high and low expression groups. |log FC|>2 and DEG with FDR<0.05 were used as screening criteria. **(c)** Bubble plot for GO enrichment analysis of DEG between high and low Siglec-15 expression in TCGA-RLPS patients. **(d)** Bar plot for KEGG enrichment analysis of DEG between high and low Siglec-15 expression in TCGA-RLPS patients. **(e)** PPI Siglec-15 related DEG's PPI network and the most important network core genes. DEG's PPI network is constructed using String. A PPI pair with a minimum interaction score of 0.9 was chosen by this study to construct a PPI network. **(f)** Bar plot for network core genes with the greatest number of adjacent nodes.

The 48 cases of RLPS from TCGA were divided into two groups according to the median level of Siglec-15 mRNA expression, and there was no significant difference in DFS (*P*=0.417) and OS (*P*=0.591) between these two groups (Figure 1A). Next, survival Analysis of GEPIA was used to analyze the prognostic value of Siglec-15 in 259 sarcoma patients, wherein the median expression of Siglec-15 was used as the cut-off value of the two groups. High expression of Siglec-15 was associated with poorer OS (*P*=0.034) of sarcoma patients (Figure S1A). And there was no significant difference in DFS between these two groups (P=0.11) (Figure S1A).

### Identification and enrichment analysis of DEGs associated with Siglec-15 expression in RLPS and sarcoma

According to the FDR value and logarithmic fold change criteria, a total of 682 differential genes between high and low Siglec-15 expression groups in RLPS were screened. Figure 1B showed the DEG heatmap. Enrichment analysis of the KEGG pathway showed that Siglec-15 was related to the Hippo signaling pathway and neuroactive ligand-receptor interaction etc. (Figure 1C). GO annotation analysis showed that the expression of Siglec-15 might affect serine hydrolase activity, and amine transport etc. (Figure 1D). Figure 1E showed the PPI protein network was composed of different genes between these two groups. The top 5 protein genes with the largest number of connection points were APOA1, F2, AHSG, AMBP, SERPINC1 (Figure 1F). Figure S1 showed the results of the constructed heatmap, KEGG enrichment analysis and GO function annotation of 259 cases of sarcoma tissues grouped according to Siglec-15 expression level.

### Siglec-15 expression was negatively correlated with PD-L1 expression

**Figure 2:**
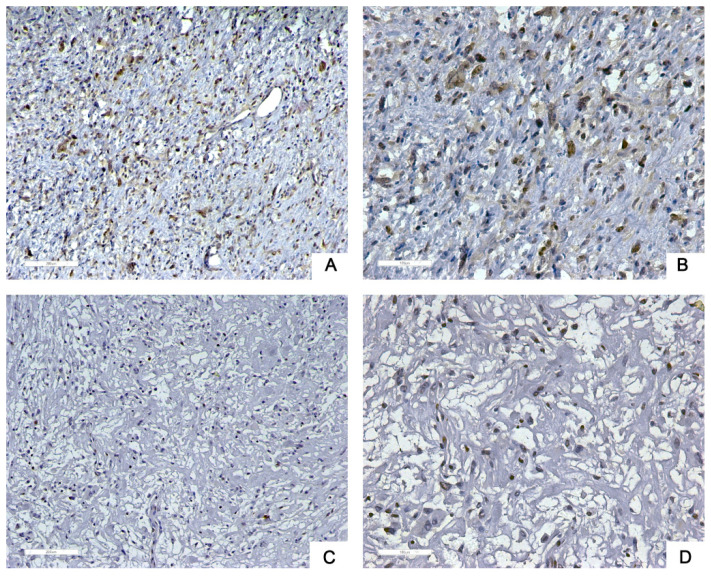
** Typical immunohistochemical staining of Siglec-15 in retroperitoneal liposarcoma. (a)** Positive Siglec-15 expression. (100× magnification); **(b)** positive Siglec-15 expression (200× magnification); **(c)** negative Siglec-15 expression (100× magnification); **(d)** negative Siglec-15 expression (200× magnification). Siglec-15 was typically located in the cytoplasm.

**Figure 3:**
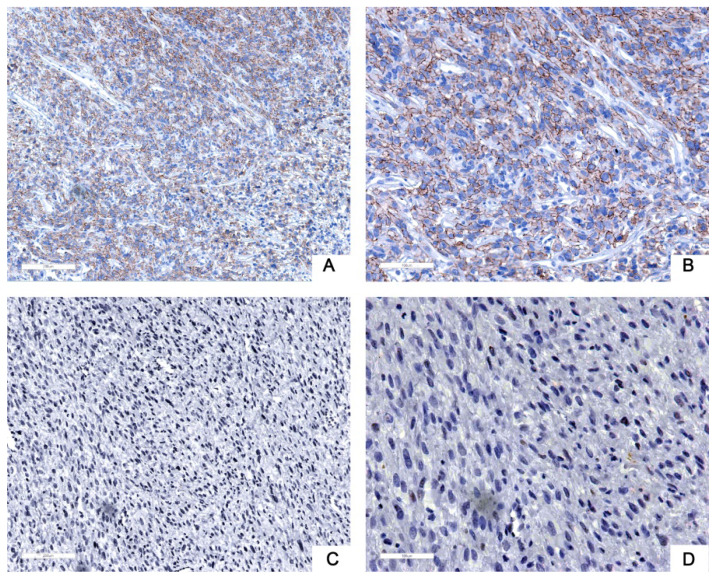
** Typical immunohistochemical staining of PD-L1 in retroperitoneal liposarcoma. (a)** Positive PD-L1 expression (100× magnification); **(b)** positive PD-L1 expression (200× magnification); **(c)** negative PD-L1 expression (100× magnification); **(d)** negative PD-L1 expression (200× magnification). PD-L1 was mainly located in the cytoplasm and on the membrane.

Next, we used tissue samples from 91 patients who accepted RLPS resection in our center to further verify the bioinformatics analysis results. Siglec-15 and PD-L1 expression was assessed using immunohistochemistry (IHC) in this study. Typical IHC staining for these two proteins was shown in Figure 2 and Figure 3, respectively. Siglec-15 was typically located in the cytoplasm, whereas PD-L1 was mainly located in the cytoplasm and on the membrane.

**Figure 4:**
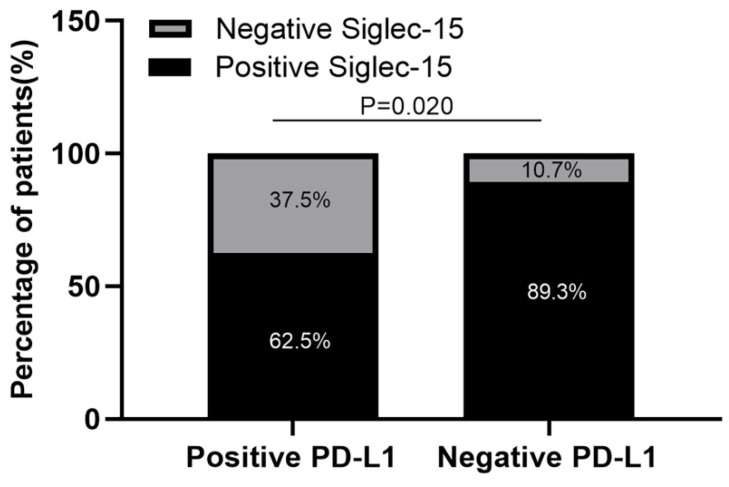
** Siglec-15 expression was negatively correlated with PD-L1 expression in RLPS patients.** 89.3% (67/75) of PD-L1-negative RLPS patients expressed Siglec-15, whereas 62.5% (10/16) of PD-L1-positive RLPS patients expressed Siglec-15. Siglec-15 expression was negatively correlated with PD-L1 expression (*r*=-0.283; χ^2^= 5.378; P=0.020).

Of the 91 specimens collected from our center, 77 were Siglec-15 positive, whereas only 16 were PD-L1 positive (84.6% versus 17.6%, respectively), 10 (11.0%) specimens were positive for both PD-L1 and Siglec-15, 8 (8.8%) were double negative, 67 (73.6%) were only positive for Siglec-15, and 6 (6.6%) were only positive for PD-L1 (Table 3). High expression of Siglec-15 was detected in 52 (57.1%) specimens, whereas high expression of PD-L1 was detected in only 7 (7.7%) specimens; 89.3% (67/75) of PD-L1-negative patients expressed Siglec-15, whereas 62.5% (10/16) of PD-L1-positive patients expressed Siglec-15 (Figure 4). Moreover, the χ^2^ test showed that there was a negative correlation between PD-L1 and Siglec-15 expression (*r*=-0.283 *P*=0.020, Table 3).

### Correlation between Siglec-15 and PD-L1 expression levels and clinicopathological features in patients with RLPS

The association between Siglec-15 expression and clinicopathological characteristics was assessed in RLPS. Among the 91 RLPS cases collected from our center, low Siglec-15 expression was associated with necrosis (*P*=0.035) (Figure S2). However, the relationship between Siglec-15 expression and sex, age, tumor size, FNCLCC grade, histopathological classification, multifocality, and recurrence was not significant. In addition, positive Siglec-15 expression was associated with age (*P*=0.036) and multifocality (*P*=0.006) (Table S1).

We also assessed the correlation between PD-L1 expression levels and clinicopathological features of patients with RLPS. As shown in Table 2, among the 91 RLPS cases, high PD-L1 expression was associated with recurrence (*P*=0.049) (Figure S3). In contrast, the relationship between high PD-L1 expression and sex, age, tumor size, FNCLCC grade, histopathological classification, multifocality, or necrosis was not significant. On the other hand, positive PD-L1 expression was associated with tumor size (*P*=0.012) (Table S2).

### Correlations of Siglec-15 and PD-L1 expression levels with survival of RLPS patients

**Figure 5:**
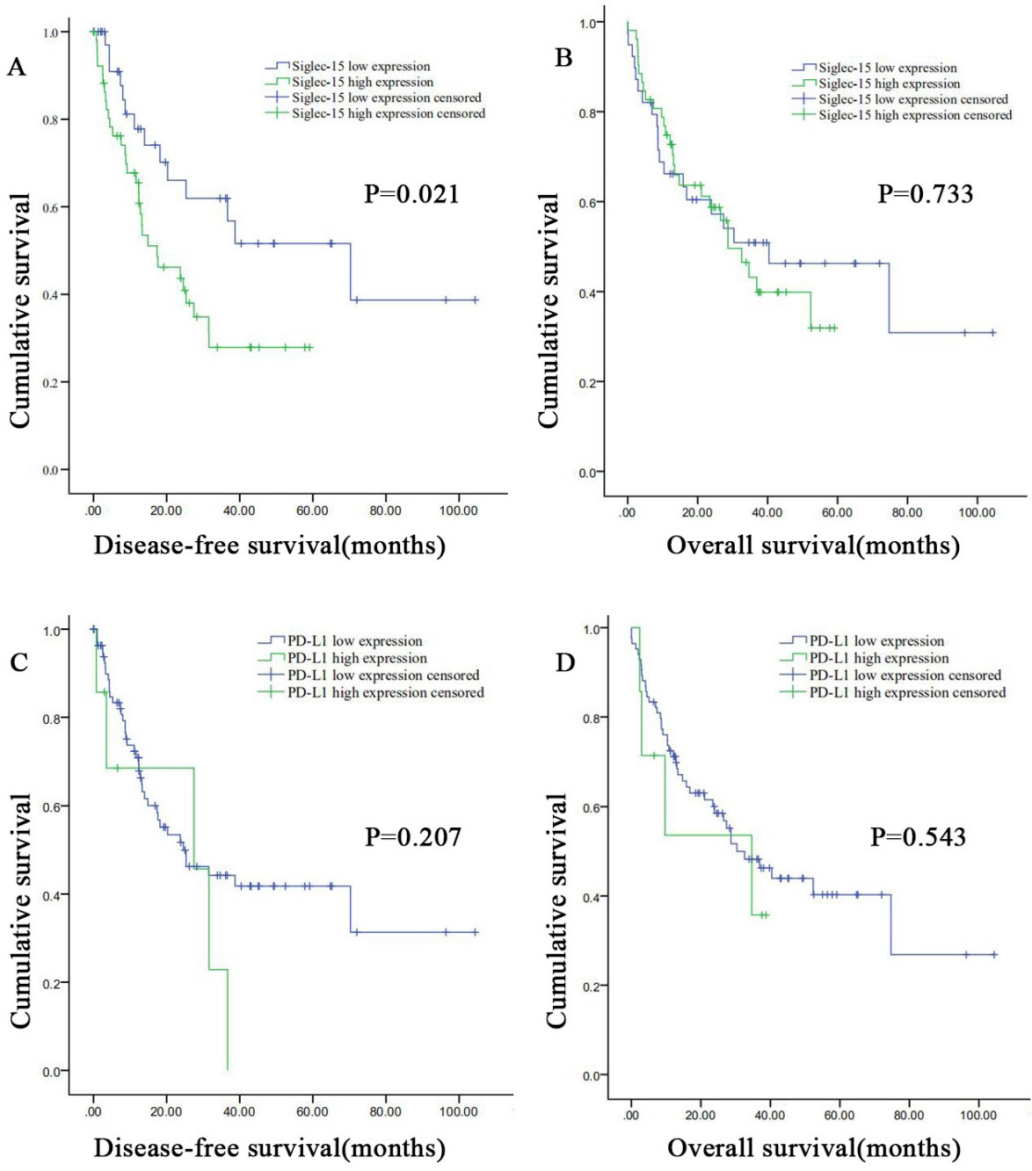
** Correlation between Siglec-15 or PD-L1 expression levels and prognosis of patients with retroperitoneal liposarcoma.** According to Siglec-15 and PD-L1 expression level, all the malignant pancreatic tumor patients were divided into high expression group and low expression group. High Siglec-15 expression was associated with poorer DFS in retroperitoneal liposarcoma patients. Kaplan-Meier survival curves for **(a)** disease-free survival (DFS) and **(b)** overall survival (OS) between patients with high and low Siglec-15 expression. Kaplan-Meier survival curves for **(c)** DFS and **(d)** OS between patients with high and low PD-L1 expression.

The DFS and OS survival curves according to the PD-L1 and Siglec-15 expression are shown in Figure 5. As for Siglec-15, Kaplan-Meier survival analysis and log-rank test showed that high Siglec-15 expression was associated with poorer DFS of RLPS patients. The median DFS time was 17.4 ± 6.4 months and 70.3 ± 25.2 months in the high and low Siglec-15 expression groups, respectively (*P*=0.021). On the other hand, the median OS time was 28.7 ± 5.0 months and 40.4 ± 17.0 months in the high and low Siglec-15 expression groups, respectively, but there was not a significant correlation (*P*=0.733). In addition, no significant difference was observed in DFS (*P*=0.095) and OS (*P*=0.150) between the positive and negative Siglec-15 groups (Figure S4). As for PD-L1, the median DFS time was 27.5 ± 13.8 months and 24.6 ± 7.5 months in the high and low PD-L1 expression groups, respectively, with no significant difference (*P*=0.207). On the other hand, the median OS time was 34.7 ± 17.6 months and 30.3 ± 6.9 months in the high and low PD-L1 expression groups, respectively, with no significant difference (*P*=0.543). Furthermore, no significant correlation in DFS (*P*=0.485) and OS (*P*=0.286) was observed between the positive and negative PD-L1 groups (Figure S4).

To determine whether combined expression of PD-L1 and Siglec-15 might affect survival, we classified all patients into two groups: PD-L1+/Siglec-15+ group and all other expression statuses as the other group. However, no significant difference was observed in DFS (*P*=0.101) and OS (*P*=0.058) between the PD-L1+/Siglec-15+ group and the other group.

Next, a Cox proportional hazard model was used to test whether clinicopathological features might affect OS and DFS in RLPS. Univariate analysis showed that high Siglec-15 expression (hazard ratio [HR]: 2.120; 95% confidence interval [CI]: 1.105-4.068; *P*=0.024), recurrence (HR: 1.883; 95% CI: 1.031-3.438; *P*=0.039), and tumor size (*P*=0.019) were significantly associated with DFS. Because few risk factors were identified, we included the risk factors with *P* < 0.2 into the multivariate regression model, which showed that high Siglec-15 expression (HR: 2.298; 95% CI: 1.154-4.576; *P*=0.018), necrosis (HR: 2.052; 95% CI: 1.082-3.890; *P*=0.028), and the tumor size (*P*=0.049) were independent predictive factors for DFS (Table 4). On the other hand, univariate analysis showed that the FNCLCC grade (HR: 2.473; 95% CI: 1.040-5.881; *P*=0.041), necrosis (HR: 1.835; 95% CI: 1.028-3.274; *P*=0.040), and tumor size (*P*=0.027) were significantly associated with OS (Table 5). Multivariate analysis revealed that necrosis (HR: 1.806; 95% CI: 1.002-3.254; *P*=0.049) and tumor size (*P*=0.021) were independent predictive factors for OS as shown in Table 5.

## Discussion

RLPS is a type of rare malignancy for which surgical resection is the primary treatment. There are limited options for recurrent and advanced sarcomas, so immunotherapy and targeted therapy are urgently needed. A few clinical trials of immunotherapy on sarcoma patients have reported positive results 5, 30, 31. Immune biomarkers such as PD-1/PD-L1 and Siglec-15 can facilitate our understanding of the immune status of sarcomas and may help explain the reasons underlying the differences in immunotherapy response. Moreover, Siglec-15 is considered to play an important role in immune escape, and it might be an important supplementary pathway to the PD-1/PD-L1 pathway 19. Hence, our study further clarified the correlation between these two typical biomarkers and their correlation with prognosis in RLPS patients.

The PD-1/PD-L1 checkpoint pathway is the most widely used target in immunotherapy. PD-1 is a transmembrane protein expressed on T cells and can regulate immune escape 32. PD-L1 is one of the well-studied ligands for PD-1 and has been detected in immune, malignant, and stromal cells in previous studies 33. In addition to its immunomodulatory effect, PD-L1 has also been used as a predictor for prognosis and immunotherapy response 34, 35. Several reports have shown that the copy number and expression level of PD-L1 are related to poor survival in sarcoma cases 7. Nevertheless, the predictive value of PD-L1 varies among STS subtypes 1, 15. In our study, PD-L1 was not a predictor of OS (P=0.543) and DFS (P=0.207) in RLPS patients, and only 16 of 91 specimens (17.6%) were PD-L1 positive. This is also in accordance with the slower development of PD-L1 clinical trials for STS. In another study, the positive expression rate of PD-L1 in smooth muscle is as high as 59%. 36. The different PD-L1 expressions among STS patients indicate that it is necessary to identify the potential beneficiary patients. Moreover, due to the lower PD-L1 expression in RLPS patients compared with other STS patients, it is extremely important to explore new immunotherapy markers that can complement PD-L1 treatment for RLPS.

Siglec-15 was previously characterized as a type I transmembrane protein with a sialic acid-binding site. Unlike most proteins in the Siglec family that have multiple C2-set Ig domains, Siglec-15 has only one IgC2 domain, which is similar to the vast members of the B7 family. In addition, its high homology with PD-L1 indicates its unique molecular features and highlights its possible correlation with the PD-L1/PD-1 pathway 19. As our study has confirmed, there was a negative relationship between PD-L1 and Siglec-15 expression (*r*=-0.283, *P*=0.020). A previous study has demonstrated that Siglec-15 and PD-L1 are mutually exclusive in lung cancer 18. Moreover, *in vivo* and *in vitro* experiments have shown that inhibition of Siglec-15 function using monoclonal antibodies (mAbs) can play a synergistic effect with anti-PD1 mAbs in colon cancer 19. Taken together, these findings indicated that the immune regulation of Siglec-15 is independent of the PD-1/PD-L1 pathway.

Siglec-15 has been originally identified as an important regulator of osteoclast development and differentiation 37. Abnormal expression of Siglec-15 was closely related to autoimmune diseases and cancer 38. In contrast with the limited expression in normal tissues, Siglec-15 mRNA expression was upregulated in a variety of tumors, which was found through analyses of TCGA database 19, 23. Li et al. also reported that higher Siglec-15 mRNA levels predicted poorer relapse-free survival (RFS) by pan-cancer analysis 23. Similar to the results of Li et al., our study confirmed that high expression of Siglec-15 was detected in 57.1% RLPS specimens. Meanwhile, our study also showed that Siglec-15 can be used as a prognostic indicator, wherein high Siglec-15 expression was an independent predictive factor for DFS. Consistent with the data of 259 sarcomas found within the TCGA database, the high expression of Siglec-15 was associated with the poorer DFS in the 91 RLPS patients in our study. However, there is no obvious correlation between Siglec-15 expression and DFS in the 48 patients with RLPS collected from the TCGA database. Compared and contrast with the TCGA database, we have expanded the number of retroperitoneal liposarcoma patients, which may also result in the inconsistent results. Furthermore, in this study, though only 17.6% of cases were positive for PD-L1 expression in RLPS patients, 84.6% of the cases were positive for Siglec-15 expression. Compared with the low expression of PD-L1, which may limit the immunotherapy effect in some patients, the high expression of Siglec-15 in RLPS patients suggests that Siglec-15 might act as a potential target of immunotherapy and as an important supplement to PD-L1 therapy. All these results suggest that compared with slow progression in PD-1 pathway, Siglec-15 might be a more suitable target for immunotherapy of sarcomas which were previously considered as immune desert tumors.

A recent study found that Siglec-15 can be expressed in tumor cells and tumor associated macrophages, and they confirmed that macrophage-associated Siglec-15 could suppress antigen-specific T cell responses. In addition, anti-Siglec-15 mAbs can also inhibit tumor growth of MC38 cells in which Siglec-15 was overexpressed 19. Hence, the function of Siglec-15 in tumor cells also needs extensive elucidation. Our study showed that low Siglec-15 expression was associated with necrosis (P=0.035), indicating that Siglec-15 might play an important role in RLPS proliferation. At present, the molecular mechanism by which Siglec-15 affects proliferation remains unclear. Mice lacking Siglec-15 had no obvious physical abnormalities except mild bone disease 39, 40. NC318, an anti-Siglec-15 mAb, is undergoing a phase I clinical trial, and its good safety and tolerability has encouraged the development of phase 2 clinical trials to evaluate its efficacy 18. GO enrichment of the high-throughput sequencing data of 48 RLPS patients from TCGA suggested that Siglec-15 expression was related to serine hydrolase activity. MDM2 oncogene amplification is the main molecular feature of well-differentiated and de-differentiated liposarcoma 41. Cissé MY et al. reported the combination of MDM2 and chromatin could mediate serine metabolism, and would thereby regulate the proliferation of liposarcoma 42. Our GO enrichment analysis showed that Siglec-15 was related to serine metabolism, which also indicated that Siglec-15 might play a key role in the development and progression of retroperitoneal liposarcoma in addition to immunity. The correlation between differential genes in sarcoma and lipid metabolism-related pathways (PPAR) provided evidence for the close relationship between Siglec-15 and liposarcoma 43. In subsequent studies, we will pay further attention to the effect of Siglec-15 upon the development and progression of liposarcoma, in addition to its effect on immunity.

In addition, KEGG enrichment analysis showed that the Hippo signaling pathway is closely related to the expression of Siglec-15 in RLPS. The Hippo signaling pathway can play an important role in tumor immunity 44, 45. Toshiro Moroishi et al. established three different mouse homologous tumor models (B16, SCC7 and 4T1), which confirmed that the lack of Hippo pathway kinase LATS1/2 (large tumor suppressor 1 and 2) in tumor cells could improve tumor immunogenicity, thereby enhancing anti-tumor immune response and inhibit tumor growth 46. Janse van Rensburg HJ et al identified the immune checkpoint molecule PD-L1 as the target of the Hippo signaling pathway. TAZ, a component of the Hippo pathway, promotes immune escape of human cancer cells through the transcriptional regulation of PD-L1 47. This could also explain the mutually-exclusive relationship between Siglec-15 and PD-L1.

To the best of our knowledge, this is the first study to describe the relationship between Siglec-15 and PD-L1 expression and verify the significant prognostic value of Siglec-15 in RLPS. In addition, we also analyzed the possible biological function of Siglec-15 on RLPS through the data collected from TCGA. Owing to the rare incidence of retroperitoneal liposarcoma, the number of patients in our study is relatively small. In future studies, we will include more patients and follow up for a longer period to ensure more reliable and accurate results. Currently, the specific interaction mechanism between PD-L1 and Siglec-15 is unclear; hence, functional experiments and mechanism exploration will be conducted in the future.

## Conclusions

There was a significantly negative association between Siglec-15 and PD-L1 expression in RLPS patients. Moreover, high Siglec-15 expression was a negative independent predictive factor for the prognosis of RLPS. In addition, the key pathways for Siglec-15 to regulate RLPS may be the Hippo signaling pathway, neuroactive ligand-receptor interaction, and the neuroactive ligand-receptor interaction. Further research is needed to clarify the biological effects and possible clinical application of Siglec-15.

## Supplementary Material

Supplementary figures and tables.

## Figures and Tables

**Table 1:** Association of high Siglec-15 expression with the clinicopathological characteristics of 91 patients with retroperitoneal liposarcoma

Characteristics	Total	High Siglec-15 expression (%)	Low Siglec-15 expression (%)	*P* value
**Gender**				
Male	52	31(59.6)	21(40.4)	0.582
Female	39	21(53.8)	18(46.2)	
**Age**				
≤60	55	30(54.5)	25(45.5)	0.536
>60	36	22(61.1)	14(38.9)	
**Tumor size**				
≤15	16	11(68.8)	5(31.2)	0.301
15-30	50	25(50.0)	25(40.0)	
>30	25	16(64.0)	9(36.0)	
**FNCLCC Grade**				
Low (G1)	20	12(60.0)	8(40.0)	0.770
High (G2, G3)	71	40(56.3)	31(43.7)	
**Histology**				
DDLPS	61	34(55.7)	27(44.3)	0.682
WDLPS	21	14(66.7)	7(33.3)	
PLPS	7	3(42.9)	4(57.1)	
MLPS	2	1(50.0)	1(50.0)	
**Multifocality**				
No	54	30(55.6)	24(44.4)	0.830
Yes	37	22(59.5)	15(40.5)	
**Recurrence**				
No	38	27(56.3)	21(43.7)	0.856
Yes	43	25(58.1)	18(41.9)	
**Necrosis**				
No	60	39(65.0)	21(35.0)	**0.035**
Yes	31	13(41.9)	18(58.1)	

**Abbreviations:** SD, standard deviation; FNCLCC, Federation Nationale des Centres de Lutte Contre le Cancer; Siglec-15, sialic acid-binding immunoglobulin-like lectin-15; WDLPS, well-differentiated liposarcoma; DDLPS, dedifferentiated liposarcoma; PLPS, pleomorphic liposarcoma; MLPS, myxoid/round cell liposarcoma.

**Table 2:** Association of high PD-L1 expression with the clinicopathological characteristics of 91 patients with retroperitoneal liposarcoma

Characteristics	Total	High PD-L1 expression (%)	Low PD-L1 expression (%)	*P* value
**Gender**				
Male	52	4(7.7)	48(92.3)	>0.999
Female	39	3(7.7)	36(92.3)	
**Age**				
≤60	55	4(7.3)	51(92.7)	>0.999
>60	36	3(8.3)	33(91.7)	
**Tumor size**				
≤15	16	2(12.5)	14(87.5)	0.051
15-30	50	1(2.0)	49(98.0)	
>30	25	4(16.0)	21(84.0)	
**FNCLCC Grade**				
Low (G1)	20	3(15.0)	17(85.0)	0.361
High (G2, G3)	71	4(5.6)	67(94.4)	
**Histology**				
DDLPS	61	5(8.2)	56(91.8)	0.270
WDLPS	21	1(4.8)	20(95.2)	
PLPS	7	0(0)	7(100)	
MLPS	2	1(50.0)	1(50.0)	
**Multifocality**				
No	54	3(5.5)	51(94.4)	0.436
Yes	37	4(10.8)	33(89.2)	
**Recurrence**				
No	38	1(2.1)	47(97.9)	**0.049**
Yes	43	6(14.0)	37(86.0)	
**Necrosis**				
No	60	5(8.3)	55(91.7)	>0.999
Yes	31	2(6.5)	29(93.5)	

**Abbreviations:** SD, standard deviation; FNCLCC, Federation Nationale des Centres de Lutte Contre le Cancer; PD-L1, programmed death ligand 1; WDLPS, well-differentiated liposarcoma; DDLPS, dedifferentiated liposarcoma; PLPS, pleomorphic liposarcoma; MLPS, myxoid/round cell liposarcoma.

**Table 3:** Association between Siglec-15 and PD-L1 expression in 91 patients with retroperitoneal liposarcoma

Groups	Siglec-15 positive	Siglec-15 negative	*P* value
PD-L1 positive	10	6	0.020
PD-L1 negative	67	8	

**Table 4:** Cox proportional hazard regression model analysis of disease-free survival in 91 patients with retroperitoneal liposarcoma

Characteristics	Univariate analysis			Multivariate analysis		
	Hazard Ratio	95% confidence interval	*P* value	Hazard Ratio	95% confidence interval	*P* value
**Siglec-15**						
Low-expression	1			1		
High-expression	2.120	1.105-4.068	**0.024**	2.298	1.154-4.576	**0.018**
**PD-L1**						
Low-expression	1					
High-expression	1.808	0.710-4.606	0.215			
**Gender**						
Male	1					
Female	0.911	0.498-1.666	0.763			
**Age**						
≤60	1					
>60	0.619	1.118-2.020	0.711			
**Tumor size**			**0.019**			**0.049**
≤15	1			1		
15-30	0.500	0.236-1.061	0.071	1.160	0.685-1.963	0.580
>30	1.303	0.597-2.842	0.506	0.605	0.398-0.919	0.018
**FNCLCC grade**						
Low (G1)	1			1		
High (G2, G3)	1.812	0.840-3.910	0.130	1.598	0.655-3.902	0.303
**Histology**			0.092			0.088
DDLPS	1			1		
WDLPS	0.303	0.118-0.776	0.013	0.301	0.110-0.820	0.019
PLPS	0.913	0.353-2.359	0.866	1.453	0.534-3.957	0.465
MLPS	<0.001	*	0.978	<0.001	*	0.980
**Multifocality**						
No	1					
Yes	1.442	0.802-2.593	0.221			
**Recurrence**						
No	1			1		
Yes	1.883	1.031-3.438	**0.039**	1.315	0.684-2.530	0.412
**Necrosis**						
No	1			1		
Yes	1.744	0.960-3.169	0.068	2.052	1.082-3.890	**0.028**

**Abbreviations:** Siglec-15, sialic acid-binding immunoglobulin-like lectin-15; PD-L1, programmed death ligand 1; NCLCC, Federation Nationale des Centres de Lutte Contre le Cancer; WDLPS, well-differentiated liposarcoma; DDLPS, dedifferentiated liposarcoma; PLPS, pleomorphic liposarcoma; MLPS, myxoid/round cell liposarcoma. * means the sample size included is too small to be estimated. The bold P value indicated significant difference.

**Table 5:** Cox proportional hazard regression model analysis of overall survival in 91 patients with retroperitoneal liposarcoma

Characteristics	Univariate analysis			Multivariate analysis		
	Hazard Ratio	95% confidence interval	*P* value	Hazard Ratio	95% confidence interval	*P* value
**Siglec-15**						
Low-expression	1					
High-expression	1.108	0.615-1.996	0.734			
**PD-L1**						
Low-expression	1					
High-expression	1.374	0.491-3.847	0.546			
**Gender**						
Male	1					
Female	1.392	0.783-2.472	0.260			
**Age**						
≤60	1					
>60	1.215	0.678-2.178	0.513			
**Tumor size**			**0.027**			**0.021**
≤15	1			1		
15-30	0.695	0.391-1.233	0.213	0.680	0.365-1.267	0.225
>30	0.815	0.538-1.236	0.336	0.799	0.522-1.224	0.302
**FNCLCC grade**						
Low (G1)	1			1		
High (G2, G3)	2.473	1.040-5.881	**0.041**	1.681	0.658-4.294	0.278
**Histology**			0.165			0.154
DDLPS	1			1		
WDLPS	0.377	0.158-0.897	0.027	0.355	0.145-0.872	0.024
PLPS	0.849	0.302-2.393	0.757	0.865	0.305-2.455	0.785
MLPS	1.439	0.195-10.606	0.721	1.324	0.177-9.913	0.785
**Multifocality**						
No	1					
Yes	1.139	0.639-2.033	0.658			
**Recurrence**						
No	1					
Yes	1.247	0.698-1.225	0.456			
**Necrosis**						
No	1			1		
Yes	1.835	1.028-3.274	**0.040**	1.806	1.002-3.254	**0.049**

**Abbreviations:** Siglec-15, sialic acid-binding immunoglobulin-like lectin-15; PD-L1, programmed death ligand 1; NCLCC, Federation Nationale des Centres de Lutte Contre le Cancer; WDLPS, well-differentiated liposarcoma; DDLPS, dedifferentiated liposarcoma; PLPS, pleomorphic liposarcoma; MLPS, myxoid/round cell liposarcoma. The bold P value indicated significant difference.

## Abbreviations

RPS: retroperitoneal soft tissue sarcoma
RLPS: retroperitoneal liposarcoma
PD-1: programmed cell death 1
PD-L1: programmed cell death ligand 1
STS: soft tissue sarcoma
Siglec-15: sialic acid-binding immunoglobulin-like lectin-15
WDLPS: well-differentiated liposarcoma
DDLPS: dedifferentiated liposarcoma
PLPS: pleomorphic liposarcoma
MLPS: myxoid/round cell liposarcoma
FNCLCC: Federation Nationale des Centres de Lutte Contre le Cancer
IRS: immunoreactivity score
OS: overall survival
DFS: disease-free survival
IHC: immunohistochemistry
HR: hazard ratio
CI: confidence interval
mAb: monoclonal antibody
RFS: relapse-free survival
KEGG: Kyoto Encyclopedia of Genes and Genomes
GO: gene ontology
PPI: protein-protein interaction
