# Supplementary material for: Expression and function of Siglec-15 in RLPS and its correlation with PD-L1: Bioinformatics Analysis and Clinicopathological Evidence
Source: Int J Med Sci. 2022 Oct 31;19(13):1977–88. doi: 10.7150/ijms.77193 (PMC9682511; doi:10.7150/ijms.77193)

Supplementary Materials

Supplementary Tables

Table S1 Association of Siglec-15 expression with the clinicopathological characteristics of 91 patients with retroperitoneal liposarcoma

Characteristics	Total	Siglec-15 positive (%)	Siglec-15 negative (%)	*P* value
**Gender**				
Male	52	41(78.8)	11(21.2)	0.078
Female	39	36(92.3)	3(7.7)	
**Age**				
≤60	55	43(78.2)	12(21.8)	**0.036**
>60	36	34(94.4)	2(5.6)	
**Tumor size**				
≤15	16	11(68.8)	5(31.2)	0.060
15-30	50	42(84.0)	8(16.0)	
>30	25	24(96.0)	1(4.0)	
**FNCLCC grade**				
Low(G1)	20	16(80.0)	4(20.0)	0.767
High(G2、G3)	71	61(85.9)	10(14.1)	
**Histology**				
DDLPS	50	41(82.0)	9(18.0)	0.931
WDLPS	23	20(87.0)	3(13.0)	
PLPS	7	6(85.7)	1(14.3)	
MLPS	11	10(90.9)	1(9.1)	
**Multifocality**				
No	54	41(75.9)	13(24.1)	**0.006**
Yes	37	36(97.3)	1(3.2)	

**Recurrence**				
No	48	42(87.5)	6(12.5)	0.420
Yes	43	35(81.4)	8(18.6)	
**Necrosis**				
No	60	51(85.0)	9(15.0)	>0.999
Yes	31	26(83.9)	5(16.1)	

SD standard deviation, FNCLCC Federation Nationale des Centres de Lutte Contre le Cancer, Siglec-15: sialic acid-binding immunoglobulin-like lectin-15, WDLPS well-differentiated liposarcoma, DDLPS dedifferentiated liposarcoma, PLPS pleomorphic liposarcoma, MLPS myxoid/round cell liposarcoma

Table S2 Association of PD-L1 expression with the clinicopathologic characteristics of 91 patients with retroperitoneal liposarcoma

Characteristics	Total	PD-L1 positive (%)	PD-L1 negative (%)	*P* value
**Gender**				
Male	52	10(19.2)	42(80.8)	0.633
Female	39	6(15.4)	33(84.6)	
**Age**				
≤60	55	11(20.0)	44(80.0)	0.454
>60	36	5(13.9)	31(86.1)	
**Tumor size**				
≤15	16	7(43.8)	9(56.3)	**0.012**
15-30	50	5(10.0)	45(90.0)	
>30	25	4(16.0)	21(84.0)	
**FNCLCC grade**				
Low(G1)	16	4(20.0)	16(80.0)	>0.999
High(G2、G3)	71	12(16.9)	59(83.1)	
**Histology**				
DDLPS	50	12(24.0)	38(76.0)	0.439
WDLPS	23	3(13.0)	20(87.0)	
PLPS	7	0(0)	7(100)	
MLPS	11	1(9.1)	10(90.9)	
**Multifocality**				
No	54	10(18.5)	44(81.5)	0.777
Yes	37	6(16.2)	31(83.8)	
**Recurrence**				
No	48	7(14.6)	41(85.4)	0.427
Yes	43	9(20.9)	34(79.1)	

**Necrosis**				
No	60	11(18.3)	49(81.7)	0.793
Yes	31	5(16.1)	26(83.9)	

SD standard deviation, FNCLCC Federation Nationale des Centres de Lutte Contre le Cancer, PD-L1 programmed death ligand 1, WDLPS well-differentiated liposarcoma, DDLPS dedifferentiated liposarcoma, PLPS pleomorphic liposarcoma, MLPS myxoid/round cell liposarcoma

Supplementary Figures

FigureS1 Prognosis value and functional enrichment analysis of DEG in sarcoma samples collected from TCGA with low and high-expressed Siglec-15. (a) Survival curve of differential Siglec-15 expression were analyzed in 259 sarcoma patients. (b) Representative heatmap of DEG between Siglec-15 high and low expression groups. |log FC|>2 and DEG with FDR<0.05 were used as screening criteria. (c) Bubble plot for GO enrichment analysis of DEG between high and low Siglec-15 expression in TCGA-SARC patients. (d) Bar plot for KEGG enrichment analysis of DEG between high and low Siglec-15 expression in TCGA-SARC patients. (e) Siglec-15 related DEG's PPI network and the most important network core genes. DEG's PPI network is constructed using String. A PPI pair with a minimum interaction score of 0.9 was chosen by this study to construct a PPI network. (f) Bar plot for network core genes with the greatest number of adjacent nodes.

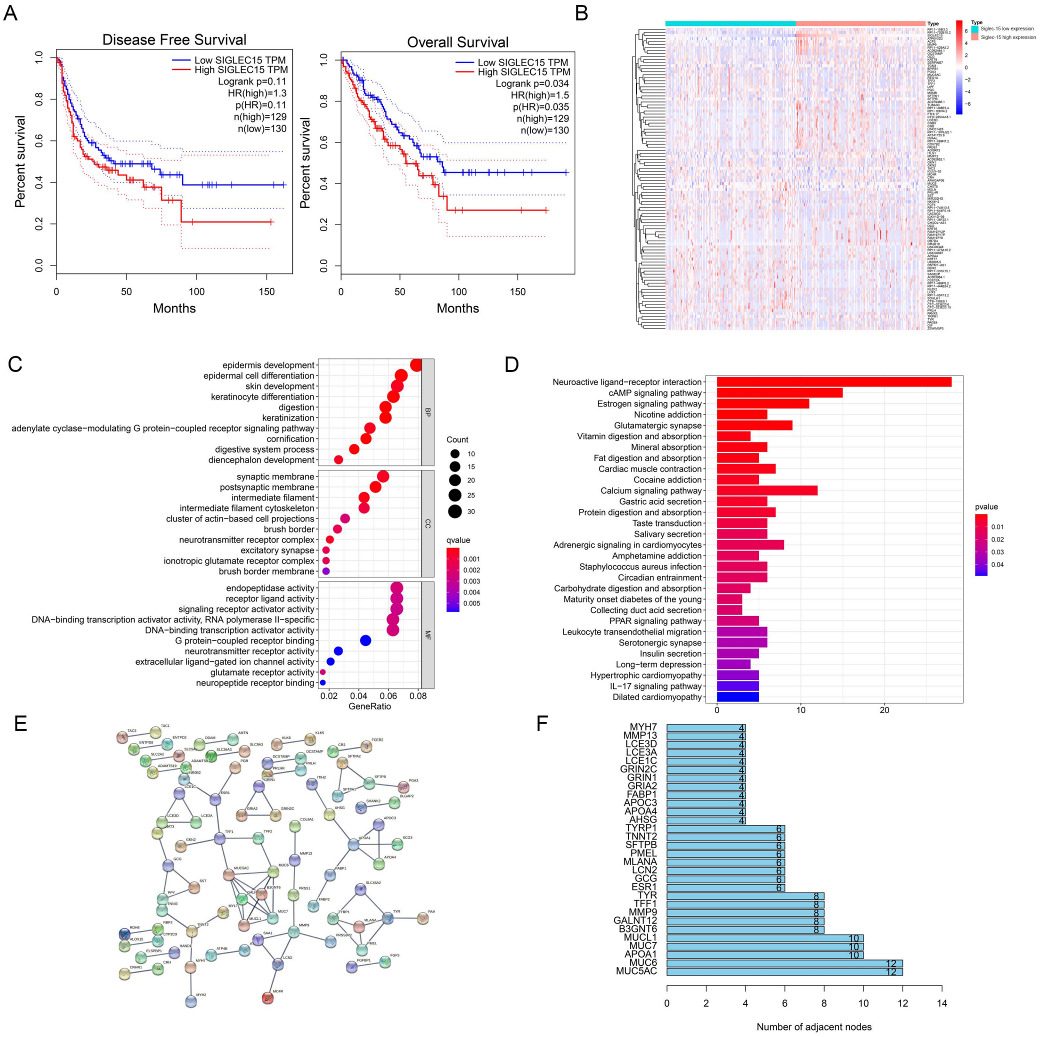

Figure S2 Low Siglec-15 expression was associated with necrosis in RLPS patients (P=0.035).

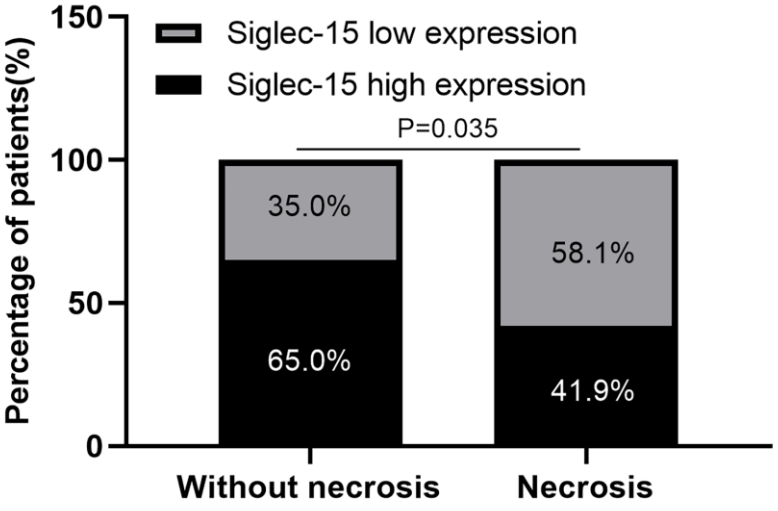

Figure S3 High PD-L1 expression was associated with recurrence in RLPS patients (P=0.049).

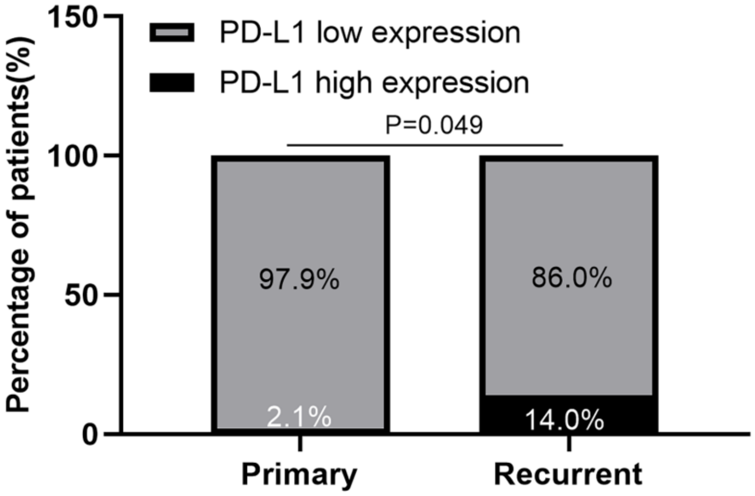

Figure S4 Correlation of PD-L1 and Siglec-15 expression with prognosis of patients with retroperitoneal liposarcoma. Kaplan–Meier survival curves for (a) disease-free survival (DFS) and (b) overall survival (OS) between patients with positive and negative Siglec-15 expression. Kaplan–Meier survival curves for (c) DFS and (d) OS between patients with positive and negative PD-L1 expression.

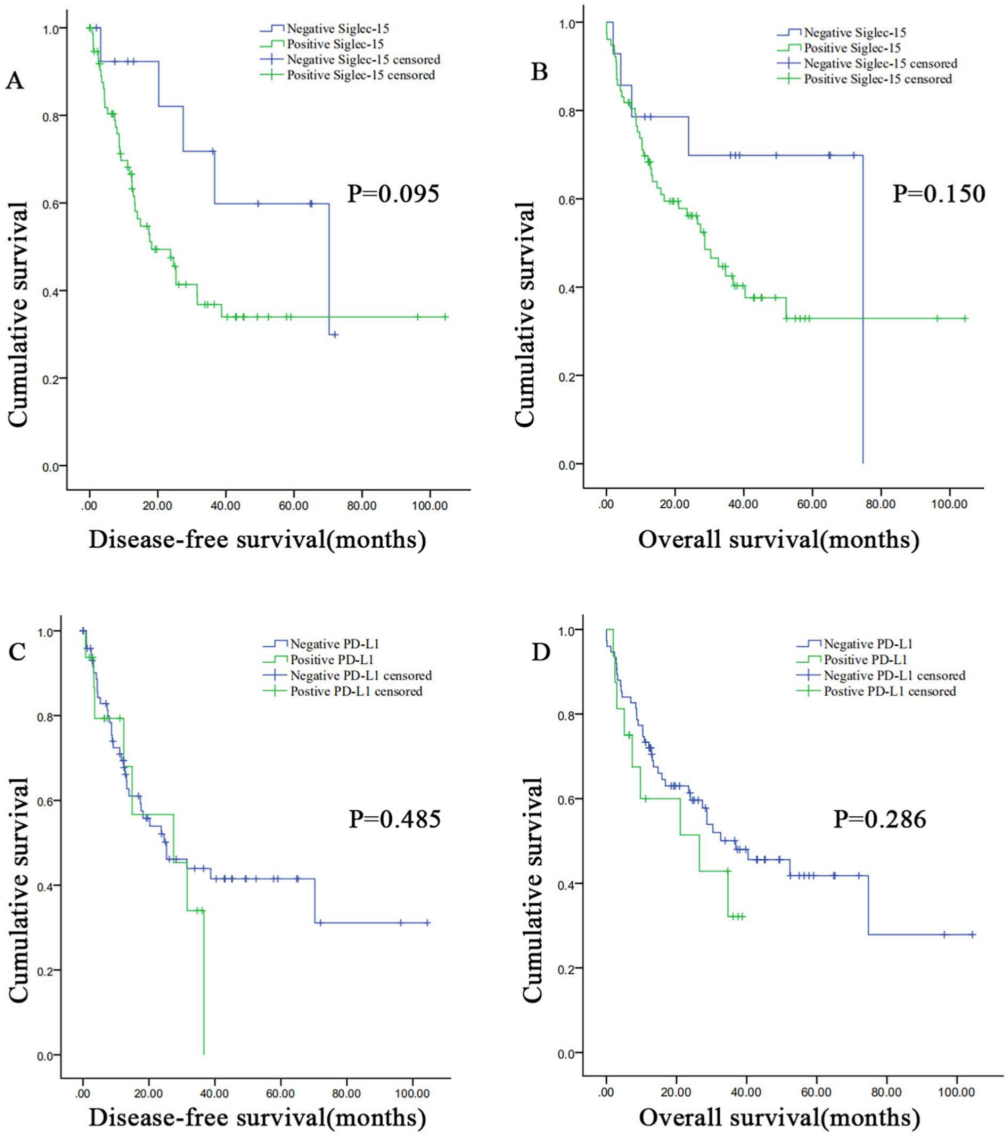

Supplement: Supplementary file 1 — Supplementary figures and tables. [file ijmsv19p1977s1.pdf]
